# Gaseous Pollutants and Particulate Matter (PM) in Ambient Air and the Number of New Cases of Type 1 Diabetes in Children and Adolescents in the Pomeranian Voivodeship, Poland

**DOI:** 10.1155/2020/1648264

**Published:** 2020-02-11

**Authors:** Małgorzata Michalska, Katarzyna Zorena, Piotr Wąż, Maria Bartoszewicz, Agnieszka Brandt-Varma, Daniel Ślęzak, Marlena Robakowska

**Affiliations:** ^1^Department of Immunobiology and Environment Microbiology, Faculty of Health Sciences, Medical University of Gdańsk, Gdańsk, Poland; ^2^Department of Nuclear Medicine, Faculty of Health Sciences, Medical University of Gdańsk, Gdańsk, Poland; ^3^Department of Pediatrics, Diabetology, and Endocrinology, Medical University of Gdańsk, Gdańsk, Poland; ^4^Department of Emergency Medicine, Faculty of Health Sciences, Medical University of Gdańsk, Gdańsk, Poland; ^5^Department of Public Health & Social Medicine, Faculty of Health Sciences, Medical University of Gdańsk, Gdańsk, Poland

## Abstract

The increase in type 1 diabetes mellitus (T1DM) incidence in children is worrying and not yet fully explored. It is suggested that probably air pollution exposure could contribute to the development of T1DM. The aim of the study was to investigate the relationship between the concentration of gaseous pollutants including, nitrogen dioxide (NO_2_), nitric oxides (NOx), sulphur dioxide (SO_2_), carbon monoxide (CO), and particulate matter (PM) in the air, and the number of new cases of T1DM in children. The number of new cases of T1DM was obtained from the Clinic of Paediatrics, Diabetology, and Endocrinology, Medical University of Gdańsk. The number of children of 0–18 years old in Pomeranian Voivodeship was acquired from the Statistical Yearbook. The concentrations of PM_10_ absorbance, NO_2_, NOx, SO_2_, and CO were measured at 41 measuring posts, between 1 January 2015 and 31 December 2016. It was detected that the average annual concentration of PM_10_ was higher than the value acceptable to the WHO. Furthermore, the average 24-hour concentration of PM_10_ was 92 *μ*g/m^3^ and was higher compared to the acceptable value of 50 *μ*g/m^3^ (acc. to EU and WHO). Moreover, the number of new cases of T1DM showed a correlation with the annual average concentration of PM_10_ (*β* = 2.396, *p* < 0.001), SO_2_ (*β* = 2.294, *p* < 0.001), and CO (*β* = 2.452, *p* < 0.001). High exposure to gaseous pollutants and particulate matter in ambient air may be one of the factors contributing to the risk of developing T1DM in children. Therefore, it is important to take action to decrease air pollutant emissions in Poland. It is crucial to gradually but consistently eliminate the use of solid fuels, such as coal and wood in households, in favour of natural gas and electricity. The development of new technologies to improve air quality, such as “best available techniques” (BAT) or renewable energy sources (water, wind, and solar generation) is of critical importance as well.

## 1. Introduction

**Figure 1:**
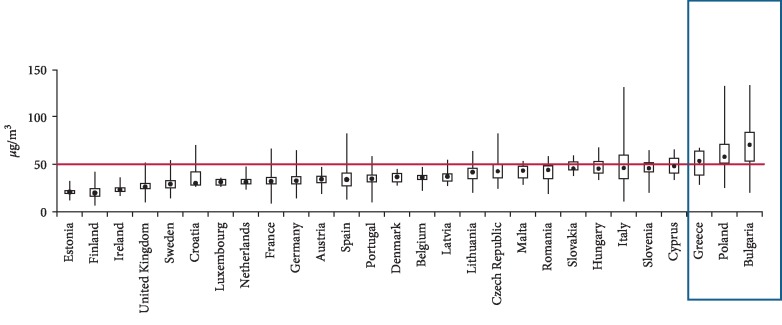
PM10 concentrations in relation to the daily limit value in 2015 in the EU. The length of the bars shows the range of reporting air quality data, with the solid black symbol indicating the mean [13].

According to the World Health Organization (WHO) air pollution contributed to 3.7 million premature deaths in 2012 globally, out of which 280000 were recorded in Europe, which constitutes a significant health problem related to environmental pollution [1, 2]. The concentration of particles with a diameter lower than 10 micrometers (PM_10_) consisting of various elements of the organic and nonorganic matter is an acknowledged indicator of air pollution. Dust particles: PM_10_–coarse particles, with a diameter below 10 *μ*m and PM_2.5_–fine particles, with a diameter of 2.5 *μ*m or less, can bond with various chemical compounds, heavy metals, or microorganisms and can be transferred over long distances, causing negative health effects [2–4]. Air pollution increases the risk of chronic obstructive pulmonary disease (COPD), lung cancer, cardiovascular diseases, strokes, allergic diseases, asthma, diabetes, and autoimmune diseases [5–9]. The source of emissions of air pollutants and suspended dust are primarily fuel combustion processes in the energy sector as well as industrial emissions associated with road transport and heating homes. In Poland, more than 88% of the energy produced comes from coal energy plants, of which nearly 53% use black coal and 35% brown coal. Atmospheric air can also be polluted by the aviation and automotive industry, which involves burning liquid fuel, the use of wearing parts in vehicles, and the rubbing of tires on asphalt surfaces [10–12]. The European Environment Agency (EEA) report on air quality in Europe in 2015 reveals that Greece, Poland, and Bulgaria are the countries where the daily PM_10_ concentration standards are exceeded [13], (Figure 1).

Dobreva et al. emphasize in their work the adverse effects of air pollution on the immune system. The authors demonstrated that air pollution, and PM_2.5_ concentrations in particular can modulate cytokine production and change the balance between Tumour Necrosis Factor *α* (TNF*α*) and the anti-inflammatory production of interleukin 10 (IL-10) in teenagers living in cities of the Stara Zagora region in the south-east of Bulgaria [14]. Research conducted in Poland showed that 5.201 asthma symptoms and 234 hospital respiratory admissions were caused annually by air pollution [15]. What's more, epidemiological studies indicate that there is a relationship between T1DM and the concentration of PM_10_ and PM_2.5_ [16–18]. Ciaula analysed data collected from 16 European countries (excluding Poland) emitting pollutants into the air and compared them with the prevalence of T1DM in children. He showed that in the investigated European countries an increase in pollution with PM_10_ corresponds to an increase in the incidence of T1DM [18]. In our preliminary studies, we showed a relationship between PM with the development of T1DM in children in the Lubelskie Voivodeship [19]. It should be added that these are the first such studies in Poland. At present, the relationship between the concentration of PM_10_, gaseous pollutants in air, and the number of new cases of T1DM in children in the Pomeranian Voivodeship was investigated.

## 2. Material and Methods

### 2.1. Geographical Location of the Pomeranian Voivodeship, Poland

**Figure 2:**
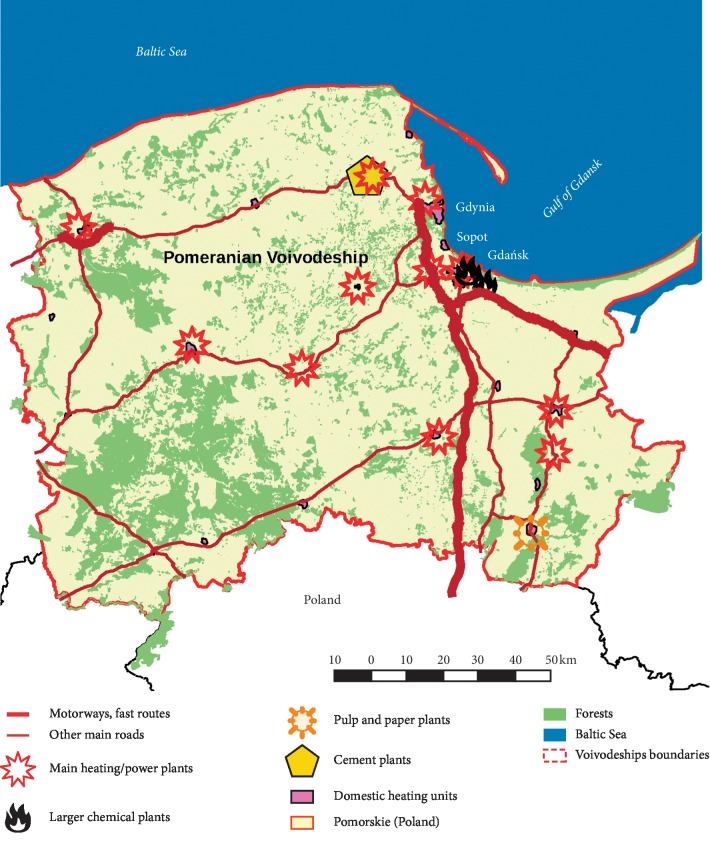
A map showing the potential gas pollution and particulate matter (PM) in the ambient air of the Pomeranian Voivodeship, Poland.

The Pomeranian Voivodeship is located in the north of Poland, on the coast of the Baltic Sea. It borders with Russia through the Gulf of Gdańsk (Figure 2). The Pomeranian Voivodeship is one of the most dynamically developing regions in central-eastern Europe. It consists of 16 poviats, including the Tricity, which is an agglomeration of Gdańsk, Gdynia, and Sopot [20, 21]. In the Tricity area, there are industrial plants producing fuels and petroleum-derived products, factories producing chemical fertilizers, feed manufacturing companies, heat and power stations, and thermal power stations and shipyards. There are also two harbours (in Gdańsk and Gdynia) which are the most important transport chain link connecting the Scandinavian countries with the countries of southern Europe [20, 21]. The natural resources in the region include vast green areas, as over 46% of the surface of the Pomeranian Voivodeship is covered by forest (Figure 2).

### 2.2. Exposure Assessment

Data on the annual average concentrations of nitrogen dioxide (NO_2_), nitric oxides (NOx), sulphur dioxide (SO_2_), carbon monoxide (CO), particulate matter, particles with a diameter of 10 micrometers or less (PM_10_), and average 24-hour concentration of PM_10_, was obtained from the Annual Evaluation of Air Quality 2015-2016, report provided by the Voivodeship Inspectorate of Environmental Protection (WIOŚ) in Gdańsk [22]. Ambient air pollution concentration measurements in Pomeranian Voivodeship were performed with the use of automatic and manual methods. To create the baseline values, the concentrations of PM_10_ absorbance, NO_2_, NOx, SO_2_, and CO were measured at 41 measuring posts for 2 years, between 1 January 2015 and 31 December 2016. NO_2_ and NOx concentrations were measured with the use of chemiluminescence, SO_2_ concentrations were measured using UV fluorescence while CO levels were measured with infrared absorption. Using polycarbonate filters, PM_10_ concentrations were determined using gravimetric analysis. The clean filters are conditioned, weighed, and placed in the collector. After 14 days, all filters were removed. In the laboratory, the filters were conditioned and weighed for the second time. The dust concentrations were calculated from the mass difference of the filter, both before and after exposure related to the air flow volume in the collector. Concentrations PM_10_, NO_2_, NOx, SO_2_, and CO are given in micrograms per cubic meter (*μ*g/m^3^). The vast majority of data series taken into consideration have completeness in excess of 75%.

In order to assess the quality of air in Poland, the country was divided into zones comprising cities of more than 100000 residents, and other areas located within the borders of the voivodeship.

### 2.3. The Incidence of Type 1 Diabetes Mellitus in the Years 2015-2016 in the Pomeranian Voivodeship

The number of new cases of T1DM was obtained from the Department of Paediatrics, Diabetology, and Endocrinology, Medical University of Gdańsk. Diabetes was diagnosed according to the Polish Diabetes Association guidelines, which correspond with the guidelines of the WHO [23, 24]. Written informed consent was obtained from all children and adolescents participating in the study, or from their parent or guardian. The study was approved by the Ethics Committee of the Medical University of Gdańsk (No. NKBBN/314/2016) and the investigation was carried out in accordance with the principles of the Declaration of Helsinki as revised in 1996.

The number of children of 0–18 years old in Pomeranian Voivodeship was acquired from the Statistical Yearbook, published by the Polish Central Statistical Office and consent was not required [25].

### 2.4. Statistical Analysis

The main procedure in the calculation is a generalized linear model–glm. An important feature of this model is the flexibility of glm function. As a consequence, extensions going beyond the simple linear models are feasible. For the data collected in the study, the Poisson error distribution and the natural logarithm as a link function have been determined. The Poisson model is similar to regular linear regression, with two exceptions. Firstly, it assumes that the errors create a Poisson distribution rather than a normal one. Secondly, instead of modelling the dependent variable (*y*) as a linear function of regression coefficients, it models the natural logarithm of the dependent variable ln (*y*).

The equation for Poisson regression is as follows:

(1) $$\begin{array}{c} \ln\left(y\right)=\beta 0+\beta x, \end{array}$$

where *β*0 and *β* are regression coefficients and *x* is an independent variable. In the considered problem, the dependent variable is the number of new cases of T1DM of children and the independent variables are one of the concentrations of gaseous pollutants.

The results have been generated using R statistics language: (A language and environment for statistical computing) version dated 2018 [26]. The assumed significance level is *α* = 0.05.

## 3. Results

### 3.1. Concentrations of Gaseous Pollutants and PM_10_ in the Air in Pomeranian Voivodeship in the Years 2015-2016

The annual NO_2_ concentrations in the Pomeranian Voivodeship were 15 *μ*g/m^3^, NOx 21 *μ*g/m^3^, SO_2_ 4 *μ*g/m^3^, and CO 374 *μ*g/m^3^ and were lower than the value acceptable to the EU and WHO [1, 13]. On the other hand, the average annual concentration of PM_10_ was 22 *μ*g/m^3^ and was higher than the value acceptable to WHO at 20 *μ*g/m^3^. Furthermore, the average 24-hour concentration of PM_10_ was 92 *μ*g/m^3^ and was higher compared to the acceptable value of 50 *μ*g/m^3^ (acc. to EU and WHO) as shown in Table 1.

### 3.2. The Number of Children Aged 0–18 Years Old in the Pomeranian Voivodeship and the Number of New Cases of T1DM

In the years 2015-2016, the number of children of 0–18 years old amounted to 947.362 in the Pomeranian Voivodeship, and the number of new cases of T1DM was 219.

### 3.3. The Relationship between the Number of New Cases of T1DM, Concentrations of Gaseous Pollutants, and Annual Average Concentration of PM_10_ in the Years 2015-2016

In the Pomeranian Voivodeship, it was found that the number of new cases of T1DM showed a relationship with the annual average concentration of PM_10_ (*β* = 2.396, *p* < 0.001) and a relationship was observed between the number of new cases of T1DM and the annual average concentration of SO_2_ (*β* = 2.294, *p* < 0.001) and CO (*β* = 2.452, *p* < 0.001). However, there was no relationship observed between either the number of new cases of T1DM and the mean annual concentration of NO_2_ (*β* = −0.010, *p* < 0.1) or the number of new cases of T1DM and the annual average concentration of NOx (*β* = −0.728, *p* < 0.1) in ambient air in the Pomeranian Voivodeship as shown in Table 2.

## 4. Discussion

The dynamic increase in the T1DM incidence in children observed in many countries, including Poland, can be associated with the growing pollution of the environment [18, 27–29]. The prevalence of T1DM in children in Poland increased 1.5 times within the 5-year observation period [27]. In order to investigate what may be the cause of the increase in the incidence of T1DM, it was decided to analyse the concentration of PM_10_ and gaseous pollutants (NO_2_, NOx, SO_2_, CO) in air and the number of new cases of T1DM in children in the Pomeranian Voivodeship. Although the annual average concentration of PM_10_ in the Pomeranian Voivodeship does not exceed the acceptable value of 40 *μ*g/m^3^ (acc. to EU), it was higher than the acceptable value recommended by WHO (20 *μ*g/m^3^). Furthermore, the maximum 24-hour concentrations of PM_10_ (*μ*g/m^3^) are higher and exceed the acceptable value of 50 *μ*g/m^3^ (acc. to EU and WHO) at each of the measuring stations located in the Pomeranian Voivodeship.

The chemical factories producing fuels and petroleum-derived products, phosphorus factories, paint factories, heat and power stations or pulp and paper plants located in the Pomeranian Voivodeship can constitute a potential source of PM_10_ and other gaseous products. Furthermore, PM_10_ emissions were mainly coming from roads with the greatest traffic volume. This pertains to the A1 highway running from Gdańsk to the South of Poland, the A7 express road to Warsaw, and the Tricity ring road [22]. The roads listed are shown in Figure 2. In addition to the higher concentration of PM_10_, the most significant source of SO_2_ in the Pomeranian Voivodeship is the emissions from fuel burning processes in the energy industry, chemical industry, and areas where the majority of houses are heated by low capacity domestic heating units. Carbon monoxide mainly comes from road transport and coal combustion in households, while NOx mainly comes from fuel burning processes in the energy industry and road transport [22].

Finally, when the Poisson regression analysis was applied, it was found that there is a relationship between the number of new cases of T1DM and the annual average concentration of PM_10_, SO_2_, and CO in ambient air in the Pomeranian Voivodeship. The current results are consistent with other authors as well as our previous results [16–19]. In 2017, Michalska et al. conducted studies in the Lubelskie Voivodeship, Poland. Poisson regression analysis showed a correlation between the number of new T1DM cases and the average annual PM10 concentration in the Lubelskie Voivodeship in 2016. However, no correlation was observed between the number of new T1DM cases and the average annual PM_10_ concentration in 2015 [19]. Hathout et al. studied cases of T1DM in children and observed a relationship between T1DM and the concentration of PM_10_ in particular in those below the age of 5 years old [16]. Beyerlein et al. showed that high exposure to the traffic-related air pollutants PM_10_, NO_2_, and possibly PM_2.5_ accelerates the manifestation of T1DM [17].

On the other hand, other findings from studies carried also indicate that particulate matter adversely affects brain structure, decreasing white matter volume or causing neuronal degeneration leading to early Alzheimer or Parkinson's disease [30, 31]. The particulate matter also increases the risk of depressive disorders and suicides [31]. The modelling results indicate that air pollution causes 2800 deaths a year in Warsaw [32]. Moreover, in epidemiological studies conducted in Cracow by Konduracka et al. a correlation was found between air pollution with PM_2.5_ and the incidence of myocardial infarction [33].

The mechanism by which air pollutants contribute to the occurrence of diseases and premature death is not fully known. The in vivo and in vitro studies conducted so far showed that even exposing healthy volunteers to pollutant particles in the air for short periods triggers an inflammatory reaction on several different levels [34–37]. The immune system recognizes the antigens via toll-like receptors that are stimulated directly or indirectly. Signal transduction, which is triggered by the receptors recognizing the antigen, activates transcription factors including Nuclear Factor Kappa B (NF*κ*B), and these in turn trigger the expression of numerous genes responsible for the production of proinflammatory chemokines and cytokines (Tumour Necrosis Factor *α*, interleukin-6) [36, 37]. Studies indicate that macrophages can capture pollutant particles and trigger an immune response in other lymphatic organs, by means of the presentation to T-lymphocytes by the dendritic cells. The organic chemical compounds contained in the dust can migrate to the systemic circulatory system and cause generalized inflammation of the vessels [4, 34, 36]. In previous studies and the studies by other authors, it was observed that patients with T1DM present with low-level inflammation, which aggravates with time and consequently contributes to the development of chronic vascular complications [36–41].

## 5. Conclusions

Considering the high level of air pollution in Poland, which exceeds the acceptable limits, and the increase in the incidence of T1DM, efforts should be made to improve the quality of air. It is believed that this can be achieved by limiting road transport in favour of rail and sea transport. In the cities, on the other hand, by replacing conventional combustion engine vehicles with electric or gas-fuelled cars and public transport. It is crucial to gradually but consistently eliminate the use of solid fuels, such as coal and wood in households, in favour of natural gas and electricity. The development of new technologies to improve air quality, such as “best available techniques” (BAT) or renewable energy sources (water, wind, and solar generation) is of critical importance as well.

## Figures and Tables

**Table 1:** Comparison of the average concentration of air pollution in the years 2015-2016 in the Pomeranian Voivodeship in Poland with the criteria used by the EU and WHO.

	Mean annual concentration of SO_2_ (*μ*g/m^3^)	Mean annual concentration of NO_2 (_*μ*g/m^3^)	Mean annual concentration of NOx (*μ*g/m^3^)	Mean 8-h concentration of CO (*μ*g/m^3^)	Mean annual concentration of PM_10_ (*μ*g/m^3^)	Mean 24-hour concentration of PM_10_ (*μ*g/m^3^)
Pomeranian Voivodeship	4	15	21	374	22	92

Acceptable concentration of pollutants (*μ*g/m^3^) acc. to the EU	20	40	30	10.000	40	50^*∗*^

Acceptable concentration of pollutants (*μ*g/m^3^) acc. to WHO	50	40	—	10.000	20	50^*∗*^^*∗*^

Abbreviations: EU: European Union; WHO: World Health Organization; SO_2_: sulphur dioxide; NO_2_: nitrogen dioxide; NOx: nitric oxide; CO: carbon monoxide; PM_10_: particulate matter 10 micrometers or less in diameter; ^*∗*^not to be exceeded on more than 35 days per year ^*∗∗*^99th percentile 3 day/year.

**Table 2:** The relationship between the number of new T1DM cases and the annual average concentration of PM10 and gaseous pollutants in the air in the years 2015-2016.

Parameter	*β*	Statistical significance
Mean annual PM10 concentration vs. the number of new T1DM cases	2.396	<0.001
Mean annual concentration of sulphur dioxide (SO_2_) vs. the number of new T1DM cases	2.294	<0.001
Mean annual concentration of carbon monoxide (CO) vs. the number of new T1DM cases	2.452	<0.001
Mean annual concentration of nitrogen dioxide (NO_2_) vs. the number of new T1DM cases	−0.010	0.1
Mean annual concentration of nitrogen oxides (NO) vs. the number of new T1DM cases	−0.728	0.1

PM_10_: particulate matter 10 micrometers or less in diameter; T1DM: type 1 diabetes mellitus; significance (*p* < 0.05).

## Data Availability

The data used to support the findings of this study are available from the corresponding author upon request.

## Abbreviations

GUS: Polish Central Statistical Office
WHO: World Health Organization
EEA: European Environment Agency
EU: European Union
T1DM: Type 1 diabetes mellitus
COPD: Chronic obstructive pulmonary disease
WIOŚ: Voivodeship inspectorate of environmental protection
PM10: Particulate matter 10 micrometers or less in diameter
PM2.5: Particulate matter 2.5 micrometers or less in diameter
PM1: Particulate matter 1 micrometers or less in diameter
SO_2_: Sulphur dioxide
NOx: Nitric oxide
NO_2_: Nitrogen dioxide
CO: Carbon monoxide
NF*κ*B: Nuclear factor kappa B.
